# Recreational Nitrous Oxide Use and Associated Neuropsychiatric Presentations in Patients Attending the Emergency Department

**DOI:** 10.3390/epidemiologia6040070

**Published:** 2025-11-01

**Authors:** Katy Boyce, Harshini M. Liyanage, Emma Tam, Soumitra Das

**Affiliations:** 1 Western Health, Melbourne 3021, Australia; katy.boyce1@wh.org.au; 2 The Faculty of Medicine, Dentistry, and Health Sciences, University of Melbourne, Melbourne 3010, Australia; tamet@student.unimelb.edu.au

**Keywords:** nitrous oxide, emergency department, neuropsychiatry manifestations, psychosis, methylmalonic acid, homocysteine, polysubstance use

## Abstract

Background/Objectives: Nitrous oxide (N_2_O), commonly known as laughing gas, is increasingly being used recreationally. While neurological risks are recognized, psychiatric effects remain underexplored. This study investigates neuropsychiatric presentations among patients referred to the Emergency Mental Health (EMH) team at Sunshine Hospital, Melbourne, Australia, associated with recreational N_2_O use. Methods: We conducted a retrospective observational review of EMH referrals between August 2020 and July 2024. Inclusion criteria were patients with documented recreational N_2_O use within the preceding 12 months. Cases were operationally defined as presenting with either predominantly psychiatric features (psychosis or suicidal ideation/self-harm documented by clinician) or predominantly neurological features (ataxia, paresthesia, pyramidal signs, or other focal deficits). Primary outcomes included type and severity of neuropsychiatric presentation, concurrent substance use, and disposition from the Emergency Department. Results: Of 25 identified patients, 23 met inclusion criteria (12 males, 11 females; mean age 29.3 ± 8.3 years). Psychotic symptoms were reported in 11/23 (47.8%, 95% CI 27.3–69.0) and suicidal ideation or self-harm in 8/23 (34.8%, 95% CI 17.2–55.7). Neurological symptoms, including paraesthesia and ataxia, occurred in 5/23 (21.7%, 95% CI 7.5–43.7). Concurrent substance use was documented in 19/23 (82.6%, 95% CI 61.2–95.0), most frequently cannabis, alcohol, and tobacco. Over half of patients (12/23; 52.2%, 95% CI 30.6–73.2) identified as culturally and linguistically diverse (CALD). Conclusions: Among EMH-referred ED patients, recreational N_2_O use is associated with a spectrum of neuropsychiatric presentations, including psychosis, suicidality, and neurological symptoms. These findings reflect clinical associations rather than causal relationships and highlight the need for early recognition, targeted assessment, and appropriate follow-up in high-risk patients.

## 1. Introduction

Nitrous oxide (N_2_O), also known as laughing gas, nitro, NOS, nangs, whippets, hippy crack or buzz, was discovered in 1772, with its psychotropic properties first being documented shortly after in 1799 [1]. While it is a well-established analgesic and widely used in the food industry for whipping cream, its psychoactive effects have led to a rise in recreational use in recent decades [2,3].

This growing trend has raised significant concern among medical and public health professionals due to the potential neuropsychiatric consequences associated with N_2_O use, including acute psychosis and vitamin B12 deficiency, which can result in neurological damage and paralysis [4]. Presentations can include a broad range of psychiatric symptoms, from anxiety and hallucinations to persecutory delusions.

The literature points to a rising number of cases, particularly among young people who are socially isolated or lacking strong familial support [2]. Anecdotal evidence also suggests an increase in psychiatric presentations to emergency departments associated with N_2_O use. Factors contributing to this trend may include ease of access, regulatory loopholes, and the widespread perceived safety amongst users [5].

Recreational use often involves releasing gas from small steel bulbs (nangs, cannisters or whippets, which often store 8 g of pressurised gas), versus larger canisters (tanks) which contain 1–3 L of gas. It is usually either inhaled via a balloon or directly from the bulb or canister. The onset of effects is rapid and users often inhale N_2_O several times.

This retrospective observational study investigates patients who presented to an Emergency Department in Melbourne, Australia, with psychiatric symptoms and concurrent recreational N_2_O use. The study aims to quantify these presentations and identify recurring themes related to demographics, psychiatric manifestations, and associated risk factors. A deeper understanding of the changing demographics of N_2_O users can develop clinical awareness of health professionals and contribute to improved patient care.

## 2. Materials and Methods

This retrospective observational study reviewed medical records of all patients presenting to the Emergency Department (ED) at Sunshine Hospital, Melbourne, who were referred to the Emergency Mental Health (EMH) team for psychiatric assessment between August 2020 and July 2024. Ethical approval was granted by the hospital Research and Ethics Review Committee in September 2024. The study adhered to the Strengthening the Reporting of Observational Studies in Epidemiology (STROBE) checklist for reporting observational research (Supplementary Materials).

### 2.1. Case Identification and Inclusion Criteria

**Figure 1:**
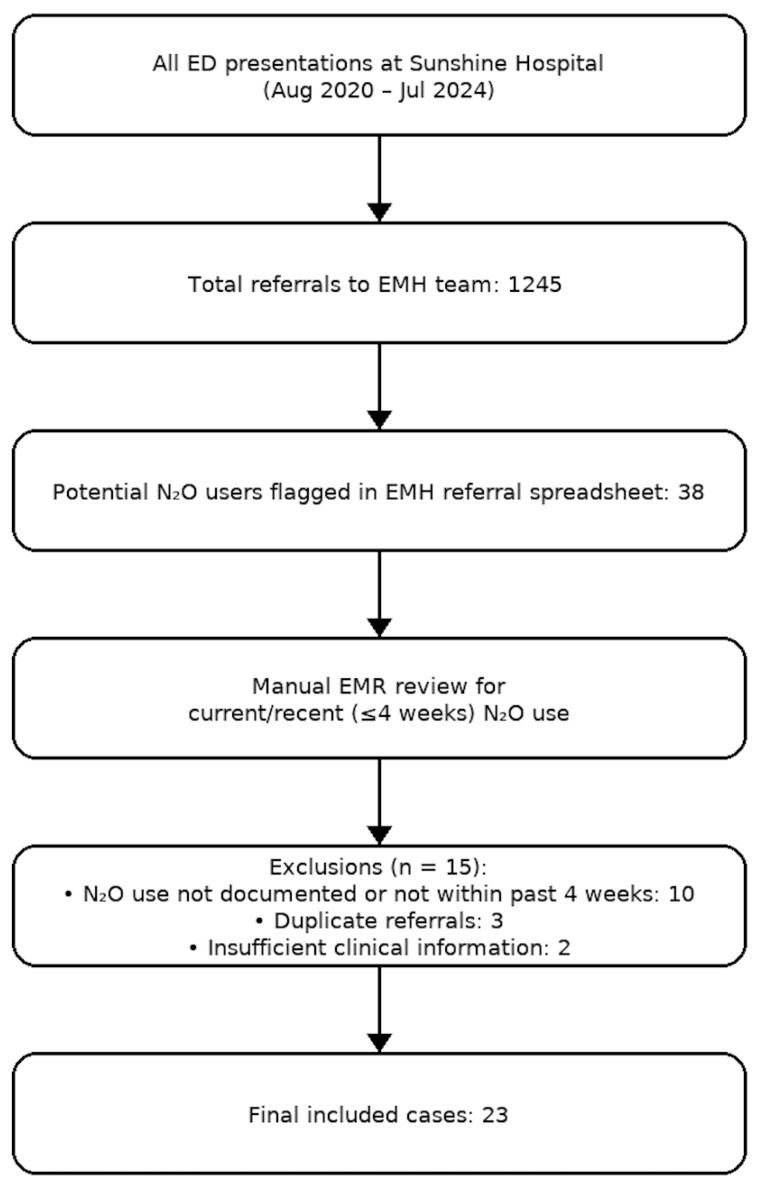
Flowchart of case identification and inclusion process for patients with N_2_O use presenting to the ED at Sunshine Hospital and referred to EMH between August 2020 and July 2024.

Patients were included if they were referred to the EMH team from the ED between August 2020 and July 2024 and had documented recreational N_2_O use within the past 12 months. The current presentation had to relate to N_2_O use, including current use or previous use within the 4-weeks. To improve clinical relevance, the exposure window was refined from “use within the past year” to “use within the last 4 weeks” prior to the index date. Cases were identified using the EMH referral spreadsheet, which logs all ED-to-EMH referrals, using keywords such as “nitrous oxide,” “N_2_O,” “whippets,” “nangs,” and “balloons.” Each potential case was manually verified via EMR review to confirm that N_2_O use was current or recent and relevant to the psychiatric or neurological presentation. Duplicate entries and incidental mentions of N_2_O not relevant to the presenting complaint were excluded. Duplicate entries and incidental mentions of N_2_O not relevant to the presenting complaint were excluded, and the overall identification and inclusion process is shown in Figure 1.

### 2.2. Prespecified Variables

The following variables were prespecified before data extraction:

- Demographics: age, sex, ethnicity (Culturally and Linguistically Diverse [CALD] status).
- Clinical features: psychiatric symptoms (psychosis, suicidal ideation/self-harm, agitation, mood disturbance), neurological signs (ataxia, paraesthesia, pyramidal signs, proprioceptive loss, Lhermitte’s phenomenon).
- Exposure characteristics: route of N_2_O administration and concurrent substance use (alcohol, cannabis, tobacco, other drugs).
- Severity classification: presentations were categorized as acute (≤7 days from N_2_O use), subacute (8–30 days), or chronic (>30 days or repeated exposure).
- Laboratory and Imaging Assessment: Where available, laboratory data included.

Vitamin B12 (assay type specified in EMR) and folate levels, on admission to ED.

Neuroimaging (MRI/CT) was performed based on documented neurological deficits, as determined by the treating team. Criteria for imaging were recorded.

- Disposition and outcomes: ED disposition (psychiatric admission, medical admission, discharge with follow-up).

### 2.3. Operational Definitions

Cases were classified as predominantly psychiatric or predominantly neurological based on the main clinical features documented at triage and/or during EMH assessment.

- Predominantly psychiatric presentations were defined as those where clinician notes or triage records documented:
  1. Psychosis (hallucinations, delusions, disorganized thought/behavior);
  2. Suicidal ideation or deliberate self-harm (DSH);
  3. Other acute mental health syndromes (e.g., severe agitation, mood episode) judged to be the primary reason for referral.
- Predominantly neurological presentations were defined as those with documented objective neurological findings, such as
  1. Ataxia or gait disturbance;
  2. Paresthesia, proprioceptive loss, or sensory changes;
  3. Pyramidal signs or Lhermitte’s phenomenon;
  4. Other focal neurological deficits judged clinically significant.

If both domains were present, classification reflected the dominant reason for referral or admission, as adjudicated by the reviewing EMH clinician.

### 2.4. Note on Standardized Instruments

No standardized psychiatric instruments (e.g., C-SSRS, structured psychosis rating scales) were used. Clinical features and outcomes were based solely on clinician-documented notes and triage records in the EMR.

### 2.5. Statistical Analysis

Descriptive statistics (means, standard deviations, frequencies, and percentages) summarized demographic and clinical characteristics. Group comparisons for continuous variables used independent samples *t*-tests, and categorical associations were tested with chi-square or Fisher’s exact tests as appropriate. Two-tailed *p*-values <0.05 were considered statistically significant. Confidence intervals (95% CI) were calculated using the Wilson method for proportions. All analyses were performed using Microsoft Excel (descriptive statistics) and R v4.3 (statistical tests).

### 2.6. Causal Language

Given the retrospective and selective EMH-referred design, findings are reported in terms of associations and clinical compatibility, avoiding causal inferences.

## 3. Results

### 3.1. Sample Characteristics

Between August 2020 and July 2024, 23 patients who presented to Sunshine Hospital Emergency Department and had documented recreational nitrous oxide (N_2_O) use were included in the analysis. Review of the data confirmed that all patients who met the original exposure definition (within the last year) had also used the medication within the previous 4 weeks prior to presentation.

The mean age was 29.3 years (SD = 8.25), with a range from 16 to 47 years. The cohort was evenly divided by gender, with 12 males (52.2%) and 11 females (47.8%) (Table 1).

Ethnicity data showed a diverse group of patients, with 12 individuals (52.2%) identifying as culturally and linguistically diverse (CALD). The most common ethnic groups were Asian (*n* = 9; 39.1%), of which Vietnamese patients made up 88.9% (*n* = 8), and Caucasian (*n* = 9; 39.1%). Other ethnicities included African (*n* = 1), Middle Eastern (*n* = 1), and Pacific Islander (*n* = 1). Two patients had unknown ethnic backgrounds (Table 1).

### 3.2. Psychiatric Presentations and Symptomatology

The most common presenting concern was psychotic symptoms, reported in 11 patients (47.8%), followed by suicidal ideation or self-harm (*n* = 8; 34.8%). Behavioural disturbances such as agitation, aggression, and confusion were present in 5 patients (21.7%), and one patient (4.3%) had thoughts of harming others (Table 2). Less frequent complaints included low mood and poor oral intake. In two cases (8.7%), the presenting complaint was not clearly documented.

Classifications of psychiatric vs. neurological were based on the operational criteria, with psychosis and suicidality forming the bulk of psychiatric cases, and gait disturbance, paresthesia, and pyramidal signs forming the majority of neurological cases.

Predominantly psychiatric presentations (*n* = 18, 78%) were differentiated from predominantly neurological presentations (*n* = 5, 22%), with psychiatric cases primarily involving psychosis or suicidality, and neurological cases presenting with paraesthesia, weakness, or ataxia.

### 3.3. N_2_O Use and Co-Substance Use Patterns

Routes of N_2_O administration varied: 9 patients (39.1%) used canisters, 3 (13.0%) used “nangs,” 2 (8.7%) used balloons, and 1 (4.3%) used a tank. Eight patients (34.8%) had undocumented methods of use. Co-occurring substance use was common, with 19 patients (82.6%) using alcohol or other drugs (AOD) alongside N_2_O, with stimulants, cannabis, tobacco products, alcohol being most common associated substances used. Only one patient (4.3%) reported N_2_O use without any other substances; data were unavailable for 3 patients (13.0%) (Table 3).

Five patients (21.7%) exhibited neurological symptoms on presentation including paraesthesia, weakness and ataxia. Serum vitamin B12 was normal in 8 patients (34.8%) and elevated in 5 patients (21.7%), with ongoing supplementation documented (type/dose/timing noted in EMR, e.g., oral cyanocobalamin 1 mg daily in 3 patients). Low B12 levels were not observed. Functional deficiency markers, including methylmalonic acid (MMA) and homocysteine, were not assessed, and this is acknowledged as a limitation. Despite the presence of neurological or neuropsychiatric symptoms, only one patient received advanced neuroimaging (MRI). This underscores a potential gap in the investigation of patients presenting with N_2_O-related complications.

### 3.4. Laboratory Results and Imaging

Serum vitamin B12 was normal in 8 patients (34.8%) and elevated in 5 patients (21.7%), with ongoing supplementation documented in EMR: oral cyanocobalamin 1 mg daily in 3 patients. Supplementation timings or duration were unclear from EMR documentation for these 3 patients. Low B12 levels were not observed. Functional deficiency markers, including MMA and homocysteine, were not assessed, and this is acknowledged as a limitation. Despite the presence of neurological or neuropsychiatric symptoms, only 1 patient (4.3%) received advanced neuroimaging (MRI) as recommended by the general medical team; this was reported as normal. This underscores a potential gap in the investigation of patients presenting with N_2_O-related complications (Table 3).

### 3.5. Risk Factors and Severity

Risk assessments identified suicidality in 8 patients (34.8%), paranoia in 6 (26.1%), and hallucinations in 5 (21.7%). One patient (4.3%) had mood-related symptoms, and another (4.3%) presented with non- psychiatric medical risk (poor oral intake). Risk factors were undocumented in two patients (8.7%) (Table 4).

Severity of presentation was classified as acute, subacute, or chronic. Acute use was more frequently associated with psychosis, while chronic presentations were associated with suicidality (Table 5).

### 3.6. Disposition and Outcome

Eight patients (34.8%) were admitted, including 7 to psychiatric inpatient units and 1 to a general medical ward. Another 8 (34.8%) were discharged with community mental health team follow-up, and 5 (21.7%) were discharged to the care of their general practitioner or family. Two outcomes (8.7%) were undocumented (Table 6).

### 3.7. Statistical Analyses

An independent samples *t*-test comparing age across two major demographic groups—Female/Asian (*n* = 11, M = 28.45, SD = 8.54) and Male/Caucasian (*n* = 12, M = 30.08, SD = 8.28)—showed no statistically significant difference (t = −0.464, df = 20.7, *p* = 0.324).

A chi-square test showed a significant association between clinical severity classification (acute, subacute, chronic) and type of presentation (suicidality vs. psychosis), χ2 (2, *n* = 70) = 21.13, *p* < 0.001. Acute cases were predominantly psychotic (72%), while chronic cases were more often suicidal (88%).

The association between gender/ethnicity and type of presentation approached significance, χ2 (1) = 3.486, *p* = 0.062, indicating a potential trend worth further study.

No significant association was observed between ethnicity and presentation severity (χ2 (4) = 3.316, *p* = 0.506), nor between ethnicity and type of psychiatric presentation (χ2 (2) = 2.931, *p* = 0.231).

## 4. Discussion

This retrospective observational study aims to explore the neuropsychiatric effects of recreational N_2_O in patients presenting to an ED in Melbourne, Australia. The findings highlight the growing clinical burden of N_2_O misuse, particularly among younger people and those engaging in concurrent substance use. Psychiatric complications, including psychosis and suicidality, were the predominant presentations, while a smaller proportion of patients exhibited neurological features. These results provide valuable insight into the demographic, clinical, and risk profiles of individuals presenting with N_2_O-related harms.

Internationally, increasing recreational N_2_O use has raised significant concern [6]. Regulatory responses vary: in the United Kingdom, N_2_O was restricted under the Psychoactive Substances Act 2016 with subsequent scheduling updates [7,8]; in the United States, sales to minors are prohibited; and in Australia, measures include labelling requirements, restrictions on sales to under-18 s, and state-level prohibitions on recreational supply. However, online availability remains widespread, and enforcement is inconsistent [3]. Including these updates provides a more current and comprehensive overview of international regulatory frameworks relevant to recreational N_2_O use.

The patients in this study had a mean age of 29.3 years, with a range from 16 to 47 years, indicating that recreational N_2_O use spans a wide age group. A predominance of younger users is consistent with surveys reporting higher N_2_O use among adolescents and young adults. Data from Western Australia showed that 18–19-year-olds were five-times more likely to report recent N_2_O use compared to their counterparts over 24-years [9]. In the UK, N_2_O is the second most used drug among 16–24-year-olds [7,8], with similar data from the US showing the use of the drug is most common between the ages of 13–39 years [5]. Social acceptance of the drug, ease of access, perceived harmlessness and low cost are likely explanations for the skew in use towards younger populations. These factors, coupled with associated experimentation with substances and risk-taking behaviours in adolescents frames N_2_O as a widely accepted mainstream drug among these populations, with a subsequently unsurprising high experimentation rate of N_2_O [10].

Gender distribution in our cohort was balanced, aligning with previous studies showing that N_2_O misuse occurs across genders [11,12,13], though some studies suggest males may be more likely to engage in frequent or high-volume use [14,15]. The near-equal representation in our sample highlights the importance of recognising N_2_O misuse as a concern not limited to one gender and underscores the need for gender-inclusive prevention and intervention strategies.

More than half of our patients identified as CALD, particularly of Asian background. While this likely reflects the demographics of Melbourne’s western suburbs, it is also consistent with emerging reports of N_2_O misuse in Asian countries where regulation remains limited [14,16]. This finding highlights the need to consider sociocultural factors in prevention strategies.

A significant proportion of the patients, 83%, had concurrent AOD use, with stimulants, cannabis and alcohol being the most commonly used substances alongside N_2_O. Such patterns complicate attribution of symptoms to N_2_O alone and present challenges for clinical management. Easy accessibility may further encourage use in social and party contexts. These observations reinforce the importance of situating N_2_O misuse within the broader landscape of polysubstance use and youth drug culture.

We operationalized ‘predominantly psychiatric’ vs. ‘predominantly neurological’ presentations using explicit criteria (psychosis, suicidality, or other acute mental health syndromes vs. documented objective neurological signs). While this improves reproducibility, some overlap exists, and cases with mixed features were classified according to the dominant presenting problem.

Psychiatric features dominated our cohort, with nearly half presenting with psychosis and more than one-third with suicidal ideation or self-harm. These acute presentations underscore the substantial mental health burden associated with N_2_O use and align with case reports describing psychosis, mood disturbance, and, in rare cases, suicide [12,17,18]. Notably, psychiatric symptoms have been reported to precede neurological complications in some cases, underscoring the importance of early identification and intervention [10,17].

There remains a paucity of robust studies specifically examining psychiatric outcomes related to N_2_O use, as many published accounts focus on vitamin B12 deficiency as the primary mechanism of neurotoxicity. While psychiatric symptoms are often mentioned in relation to B12 depletion, few studies explore their occurrence independently or in the context of polydrug use, which complicates attribution. This challenge is reflected in our sample as discussed above. The role of N_2_O as a potential gateway substance or risk amplifier in individuals already vulnerable to psychiatric illness warrants further investigation.

No cases of B12 deficiency were documented; however, serum B12 levels alone are not a fully reliable indicator of functional B12 status, as they can be influenced by prior supplementation. Functional B12 deficiency may still exist despite normal or elevated serum B12 levels, and more specific markers, such as MMA and homocysteine, are typically required to detect such cases. These markers were not measured in this study, which limits our ability to assess subclinical functional B12 deficiency. This limitation is acknowledged and highlights the need for future studies to include functional B12 markers to better understand the relationship between N_2_O use and neurological complications. 

Neurological complications were less frequent in our sample compared with neurology-led cohorts, likely reflecting the psychiatric referral pathway. 5 patients in this study reported neurological symptoms, namely paraesthesia, weakness and ataxia. These are consistent with symptoms of subacute combined degeneration of the spinal cord and peripheral neuropathy—known complications of chronic N_2_O exposure due to its inactivation of vitamin B12. Only 1 patient underwent further imaging (MRI brain and whole spine) for neurological symptoms. Given emerging data from countries such as Australia and the Netherlands, where an increase in N_2_O-related neurological presentations among young people has been noted [8], more consistent screening, such as B12 monitoring and neuroimaging, should be considered, especially in patients with a history of frequent or high-volume use.

From a clinical perspective, our findings support the need for structured assessment of N_2_O-related presentations in ED. We recommend taking a targeted history of N_2_O and other substance use, performing a focused neurological examination, and assessing vitamin B12 and folate levels, with MMA and homocysteine where available. Neuroimaging should be considered in patients presenting with neurological deficits, and early administration of hydroxocobalamin is advised in cases of suspected functional B12 deficiency. Incorporating these measures may facilitate timely diagnosis, prevent progression of neurological injury, and guide appropriate psychiatric and medical management.

Finally, clinical outcomes in our cohort varied, with one third of patients (*n* = 8) being admitted to the hospital (7 under psychiatric care and 1 under general medicine), while another 8 were discharged with follow-up by community mental health teams. 5 patients were discharged with care instructions from their general practitioners. These results highlight the need for tailored care approaches, as the severity of the psychiatric and neurological symptoms may vary significantly between individuals.

### 4.1. Limitations

This study has several limitations that should be considered when interpreting the findings. As a retrospective model, the study is subject to reporting bias and potential omissions of relevant clinical information. Accurate identification of N_2_O use depended on patient disclosure and clinician recording, which may have led to underreporting. Details such as quantity, frequency, and duration of use were often missing, limiting assessment of dose–response relationships and usage patterns. Similarly, neurological symptoms may have been incompletely documented, as these were not consistently asked in the emergency setting. Given that the sample was drawn from psychiatric referrals, neurological sequelae may have been underrecognised at initial presentation.

The relatively small sample size (*n* = 23) and single-centre design restrict the generalisability of the results. Although sample sizes in other early studies of emerging substance use have also been modest, larger and multicentre cohorts are needed to strengthen external validity. The focus on a culturally diverse region of Melbourne provides valuable insight, but findings may not fully reflect patterns of N_2_O use in the broader Australian or international context.

Case ascertainment relied on keyword searches of the EMH referral spreadsheet with subsequent EMR validation and manual confirmation. While this approach ensured systematic capture of EMH-referred cases, it may have missed patients in whom N_2_O use was not documented, coded differently, or managed solely by non-psychiatric teams.

The selective referral pathway and small sample size highlight the need for caution when extrapolating these findings to all ED presentations related to N_2_O use. Future prospective studies with broader capture of ED presentations, standardized exposure assessment, functional B12 assays, and systematic imaging would improve reliability and generalizability.

Limitations in clinical investigation may have contributed to under-detection of neurological complications. Neuroimaging was rarely performed, and vitamin B12 assessment was inconsistent, with functional markers (MMA, homocysteine) not measured. This likely underestimated the true burden of neurological injury. Documentation of treatment interventions was also inconsistent, limiting insight into effective pharmacological or psychosocial management strategies.

Finally, the retrospective nature of data collection prevented accurate temporal analysis of exposure and symptom onset. Future research should use prospective designs to systematically capture N_2_O use (dose, frequency, duration, and context), include comprehensive neuropsychiatric assessments, and collect objective biomarkers of exposure. Larger, multicentre studies incorporating demographic and psychosocial variables—such as socioeconomic status, mental health history, and social media influences—would further inform prevention and intervention strategies.

### 4.2. Implications for Future Research

The findings of this study, alongside its limitations, point to several important directions for future research and public health action. There is a clear need for prospective, multi-centre studies using standardised tools to characterise recreational N_2_O use and its neuropsychiatric effects. Longitudinal research is particularly important to clarify the chronicity of symptoms, dose–response relationships, and the potential benefits of early intervention. Including control groups in future work would allow direct comparisons with non-users and strengthen evidence on the unique contribution of N_2_O to psychiatric and neurological outcomes.

From a public health perspective, the rising prevalence of N_2_O use among young people demands immediate attention. Its low cost, ease of access, and widespread social acceptance contribute to underestimation of harm. Public health campaigns should aim to increase awareness of neuropsychiatric risks, dispel misconceptions about safety, and promote early help-seeking, particularly among high-risk groups such as adolescents and polydrug users.

Clinically, early recognition of N_2_O-related neuropsychiatric complications in the ED is essential. A targeted history of N_2_O and other substances, focused neurological examination, and assessment of vitamin B12 and folate, supplemented with MMA and homocysteine where available, should be part of standard evaluation. Neuroimaging should be considered in cases of neurological deficit, and early hydroxocobalamin therapy is recommended when functional deficiency is suspected. Consistent clinical guidelines are needed to support frontline clinicians in managing N_2_O-related harms.

Further research should also examine sociocultural and environmental risk factors, including the role of ethnicity, socioeconomic status, and social media influences, to identify vulnerable populations. Such evidence can inform prevention strategies, evidence-based treatment protocols, and broader policy responses to mitigate the growing public health impact of recreational N_2_O use.

## 5. Conclusions

This study highlights the growing public health concern surrounding recreational N_2_O use, particularly among younger individuals and CALD populations. Our findings underscore the significant neuropsychiatric risks associated with N_2_O—including psychosis, suicidal ideation, and neurological symptoms—that may be under-recognised in emergency and psychiatric care.

To address these risks, there is a pressing need for greater clinical awareness, early screening, and improved access to mental health support. Public health strategies should prioritise harm reduction, targeted prevention, and culturally informed education efforts. Ultimately, by combining clinical vigilance with broader preventative initiatives, we can more effectively respond to the challenges posed by N_2_O use and promote safer, more informed communities.

## Figures and Tables

**Table 1:** Sample Characteristic Variables.

Variable	N (%)	95% CI
Age (mean ± SD)	29.3 ± 8.25	27–69%
Gender		
Male	12 (52%)	32–71%
Female	11 (48%)	29–68%
Ethnicity		
Asian	9 (39%)	21–61%
Caucasian	9 (39%)	21–61%
African	1 (4%)	0.2–21%
Middle Eastern	1 (4%)	0.2–21%
Pacific Islander	1 (4%)	0.2–21%
Unknown	2 (9%)	1–28%
CALD Background	12 (52%)	32–71%

**Table 2:** Clinical Presentations.

Presentation Type	N (%)	95% CI
Psychotic symptoms	11 (48%)	27–69%
Suicidal ideation/self-harm	8 (35%)	17–58%
Behavioural disturbances	5 (22%)	8–44%
Thoughts of harming others	1 (4%)	0.2–21%
Mood symptoms	1 (4%)	0.2–21%
Unknown	2 (9%)	1–28%

**Table 3:** N_2_O Use, Co-Substance Use, and Neurological Findings.

Variable	N (%)	95% CI	Notes
Cannisters	14 (61%)	1–61%	Including “nangs” and “balloons”
Tanks	1 (4%)	8–44%	
Method Unknown	8 (35%)	0.2–21%	
Habitual N_2_O Use (Greater than once per week)	15 (65%)		
Occasional N_2_O Use (Less than once per week)	5 (22%)		
Concomitant AOD use	19 (83%)	0.2–21%	Includes cannabis, alcohol, tobacco
Alcohol	10 (44%)		
Cannabis	10 (44%)		
Stimulants (cocaine, ecstasy, methamphetamine, lisdexamphetamine)	13 (58%)		
Depressants (benzodiazipines, GHB)	6 (62%)		
Hallucinogens/dissociatives (ketamine, LSD)	7 (30.4%)		
Nicotine/tobacco products (cigarettes, vapes)	9 (39%)		
N_2_O Only	1 (4%)	0.2–21%	
AOD Unknown	3 (13%)	3–34%	
Neurological Symptoms	5 (22%)	8–44%	Paraesthesia, weakness, ataxia
Vitamin B12	Normal 8 (35%) High 5 (22%) Low 0 (0%) Not measured 10 (44%)	Normal 17–58% High 8–44%	MMA/homocysteine not assessed
MRI performed	1 (4%)	0.2–21%	MRI reported as normal

**Table 4:** Risk Factors.

Risk Factor	N (%)	95% CI
Suicide Risk	8 (35%)	17–58%
Paranoia	6 (26%)	11–48%
Hallucinations	5 (22%)	8–44%
Medical risk (poor intake)	1 (4%)	0.2–21%
Unknown	2 (9%)	1–28%

**Table 5:** Severity.

Severity Classification	N (%)	95% CI
Acute	11 (48%)	45–90%
Chronic	8 (35%)	53–98%

**Table 6:** Outcomes

Outcome	N (%)	95% CI
Admitted (Psychiatry)	7 (30%)	14–52%
Admitted (General Medicine)	1 (4%)	0.2–21%
Discharged with community follow-up	8 (35%)	17–58%
Discharged to GP/home	5 (22%)	8–44%
Unknown	2 (9%)	1–28%

## Data Availability

No data except the data presented in the paper are available.

## Supplementary Materials

The following supporting information can be downloaded at: https://www.mdpi.com/article/10.3390/epidemiologia6040070/s1, STROBE Checklist for “Recreational Nitrous Oxide Use and Associated Neuropsychiatric Presentations in Patients Attending the Emergency Department”.

## Abbreviations

The following abbreviations are used in this manuscript:

N_2_O	Nitrous Oxide
EMH	Emergency Mental Health
CALD	Culturally And Linguistically Diverse
ED	Emergency Department
MR	Electronic Medical Record
AOD	Alcohol and Other Drugs
MRI	Magnetic Resonance Imaging
NDHS	National Drug Strategy Household Survey
UK	United Kingdom
US	United States
NT	Northern Territory
NSW	New South Wales
