# Supplementary material for: Recreational Nitrous Oxide Use and Associated Neuropsychiatric Presentations in Patients Attending the Emergency Department
Source: Epidemiologia (Basel). 2025 Nov 1;6(4):70. doi: 10.3390/epidemiologia6040070 (PMC12641693; doi:10.3390/epidemiologia6040070)
Supplement: Supplementary file 1 [file epidemiologia-06-00070-s001.zip › epidemiologia-3851274-supplementary.pdf]

STROBE Checklist for “Recreational Nitrous Oxide Use and Associated Neuropsychiatric Presentations in Patients Attending the Emergency Department”

Item No.	STROBE Recommendation	Section of Manuscript Where Addressed
1	Indicate the study’s design with a commonly used term in the title or the abstract.	Title, Abstract (“retrospective observational study”).
2	Provide in the abstract an informative and balanced summary of what was done and what was found.	Abstract.
3	Explain the scientific background and rationale for the investigation being reported.	Introduction, paragraphs 1–4.
4	State specific objectives, including any prespecified hypotheses.	Introduction, final paragraph.
5	Present key elements of study design early in the paper.	Methods, paragraph 1 (“retrospective observational study”).
6	Describe the setting, locations, and relevant dates, including periods of recruitment, exposure, follow-up, and data collection.	Methods, Section 2: “Materials and Methods” (Sunshine Hospital, August 2020–July 2024).
7	Clearly define all eligibility criteria and the sources and methods of selection of participants.	Section 2.1 “Case Identification and Inclusion Criteria.”
8	Explain how exposures, outcomes, predictors, potential confounders, and effect modifiers were defined.	Section 2.2 “Prespecified Variables” and Section 2.3 “Operational Definitions.”
9	Describe any efforts to address potential sources of bias.	Section 2.1 and 4.1 (limitations, manual EMR verification, exclusion of incidental mentions).
10	Explain how the study size was arrived at.	Section 3.1 (All eligible cases between 2020–2024; total N=23).
11	Explain how quantitative variables were handled in the analyses. If applicable, describe which groupings were chosen and why.	Section 2.5 “Statistical Analysis” (continuous vs. categorical variables).
12	Describe all statistical methods, including those used to control for confounding.	Section 2.5 “Statistical Analysis” (t-tests, chi-square, Fisher’s exact test, confidence intervals).
13	Report numbers of individuals at each stage of study—e.g., numbers potentially eligible, examined for eligibility,	Section 3.1 “Sample Characteristics” and Figure 1 (case flow diagram).

	confirmed eligible, included in the study, and analysed.	
14	Give characteristics of study participants (demographic, clinical, social) and information on exposures and potential confounders.	Tables 1–3; Section 3.1–3.3.
15	Indicate number of participants with missing data for each variable of interest.	Tables 1–3 (e.g., "Unknown" categories indicated).
16	Report outcomes, main results, and estimates with precision (e.g., 95% confidence intervals).	Sections 3.1–3.7; Tables 1–6.
17	Report other analyses done—e.g., subgroup analyses and sensitivity analyses.	Section 3.7 (comparative analyses by gender/ethnicity).
18	Summarize key results with reference to study objectives.	Discussion, first paragraph.
19	Discuss limitations of the study, taking into account potential sources of bias or imprecision.	Section 4.1 "Limitations."
20	Give a cautious overall interpretation of results considering objectives, limitations, multiplicity of analyses, results from similar studies, and other relevant evidence.	Section 4 "Discussion" and 5 "Conclusions."
21	Discuss the generalizability (external validity) of the study results.	Section 4.1 "Limitations" (single-centre, small sample size).
22	Give the source of funding and the role of the funders for the present study and, if applicable, for the original study on which the present article is based.	Not applicable (no funding declared).
23	Indicate ethical approval and adherence to reporting guidelines.	Methods, paragraph 1 ("Ethical approval granted… study adhered to STROBE checklist").
